# Rumen-derived lipopolysaccharide enhances the expression of lingual antimicrobial peptide in mammary glands of dairy cows fed a high-concentrate diet

**DOI:** 10.1186/s12917-016-0755-z

**Published:** 2016-06-27

**Authors:** Di Jin, Guangjun Chang, Kai Zhang, Junfei Guo, Tianle Xu, Xiangzhen Shen

**Affiliations:** College of Veterinary Medicine, Nanjing Agricultural University, Nanjing, People’s Republic of China

**Keywords:** High-concentrate diet, Lipopolysaccharide, Lingual antimicrobial peptide, Mammary gland, Cow

## Abstract

**Background:**

Long-term high-concentrate diet (HCD) feeding can cause subacute ruminal acidosis in cows and subsequently trigger systemic inflammatory and immune responses. Therefore, we conducted the present study in which twelve lactating cows installed with ruminal fistula were randomly assigned to the HCD group (forage:concentrate = 4:6, *n* = 6) or the low-concentrate diet (LCD) group (forage:concentrate = 6:4, *n* = 6) and were fed for 20 weeks. Ruminal fluid, plasma and mammary gland tissue samples were collected at week 20 for analysing lipopolysaccharide (LPS), pro-inflammatory cytokines, and immune relevant gene expression. The aim of this study was to investigate the effect of rumen-derived LPS on lingual antimicrobial peptide (LAP) synthesis and immune responses in mammary glands of lactating cows fed a HCD.

**Results:**

Compared with the LCD group, the ruminal pH was lower in the HCD group, while LPS concentrations in the rumen, lacteal artery and vein were higher. The expression of LAP, BNBD5, IL-1β, IL-6, IL-8, and TNF–α was enhanced in the HCD group. LAP protein expression was higher in the HCD group than that in the LCD group. The expression of nuclear factor kappa B (NF-кB) did not change, but was activated, as the amounts of phosphorylated NF-kB and phosphorylated inhibitory kBα increased in the HCD group compared with that in the LCD group.

**Conclusions:**

After long-term HCD feeding, rumen-derived LPS translocated to the blood stream, triggered inflammatory and immune responses and enhanced LAP synthesis via the NF-kB signalling pathway in mammary glands of lactating cows.

## Background

Long-term consumption of a high-concentrate diet (HCD), which occurs to meet the energy requirements for high milk yields, is hazardous to the health of ruminants. The consumption of a HCD causes the accumulation of organic acid and a decrease in pH values in the rumen [1]. When the ruminal pH decreases below 5.6 for more than 3 h per day, subacute ruminal acidosis (SARA) occurs [2], and free LPS is released from Gram-negative bacteria and is translocated into the circulatory system across the epithelial barrier of the gastrointestinal tract [3, 4]. Subsequently, LPS initiates systemic inflammatory responses [3, 5, 6] and host innate immune responses [7] because the circulating LPS stimulates the release of pro-inflammatory cytokines such as interleukin (IL)-1β, IL-6, and tumour necrosis factor-α (TNF-α) by monocytes, macrophages and vascular cells [8]. The release of LPS and cytokines results in enhanced secretion of several antimicrobial peptides, such as lingual antimicrobial peptide (LAP), in mammary epithelial cells (MECs) [9].

LAP, which was first found in inflamed bovine tongue epithelium [10], is a type of β-defensin that often exists in epithelial cells when inflammation occurs. Defensins contribute to innate immunity and are effective against a broad spectrum of microorganisms [11]. Other previously identified bovine β-defensins include tracheal antimicrobial peptide, bovine neutrophil β-defensin (BNBD), enteric β-defensin, and many other types of bovine β-defensins [12–15]. The localization of LAP in MECs has been reported [9]; its expression is constitutive [16], such as in healthy [17], involuted [18], *E.coli*-challenged [19] or *Streptococcus uberis-*challenged [20] mammary gland tissues. LAP is also secreted from the alveolar epithelium into milk in healthy [21] and LPS-challenged [22] udders. One study demonstrated that the level and kinetics of the mRNA concentrations of LAP in *E. coli*-challenged MECs were associated with induction time but were independent of the pathogen dose [23]. Furthermore, Liu et al. [24] demonstrated that the p65 subunit of nuclear factor-kB (NF-kB) plays a stimulatory role while C/EBPβ have a repressive function in LAP expression, and mutations of the binding sites for these two transcription factors abrogated LAP expression in MECs. Some reports also found widespread LAP mRNA expression in infected bovine digestive tract mucosal and respiratory tissues [25, 26]. These results strongly indicated that LAP plays a crucial role in the innate immune response.

The effects of a HCD on gastrointestinal tract or mammary gland health have received increasing attention [27, 28]. The relationship between LAP synthesis and immune responses in mammary glands of dairy cows intramammary stimulated by LPS or pathogens has been described previously. However, the effect of rumen-derived LPS caused by a HCD on LAP synthesis and immune responses in mammary glands of cows remains unknown. Furthermore, the mechanism of this effect remains to be clarified. Therefore, the objective of this study was to investigate the effects of rumen-derived LPS on LAP synthesis and immune responses in mammary glands of cows fed a HCD.

## Methods

### Animals, diet and experimental design

Twelve multiparous mid-lactating Holstein cows (Purchased from the Experimental Farm of Nanjing Agricultural University, average body weight, 455 ± 28 kg) fitted with ruminal fistula were randomly assigned into two groups: the high-concentrate diet (HCD) group (forage:concentrate = 4:6, *n* = 6) as the treatment group and the low concentrate diet (LCD) group (forage:concentrate = 6:4, *n* = 6) as the control group (fed for 20 weeks). Dietary ingredients and nutrient compositions, which were published previously [29], are presented in Table 1. The experimental cows were housed in individual tie-stalls at the Dairy Farm of Nanjing Agricultural University (Nanjing, China). Cows were fed three times daily at 04:00, 12:00, and 20:00 h and were allowed free access to fresh water throughout the experiment. All of the cows received LCD for 4 weeks (as an adaption period) before the formal experiment to ensure the similarity of their metabolic status.

**Table 1:** Ingredients and nutrient composition of the experimental diets

Ingredients, % of DM	Percentage (%)of ingredients (dry matter)	
	LCD^1^	HCD^1^
Corn silage	30.00	20.00
Alfalfa hay	30.00	20.00
Maize	22.78	33.6
Wheat bran	5.15	15
Soybean meal	9.81	9
Calcium phosphate dibasic	0.92	0.53
Powder	0	0.52
Salt	0.35	0.35
Premix^2^	1	1
Total	100	100
Forage:concentrate	6:4	4:6
Nutritional composition^3^		
Net energy, MJ/kg	6.32	6.74
CP, %	16	16.2
EE, %	3.96	4.15
NDF, %	37.71	31.92
ADF, %	22.75	17.55
NFC, %	32.43	40.31
Ca, %	0.9	0.8
P, %	0.45	0.45

^1^ LCD, low-concentrate diet; HCD, high-concentrate diet

^2^ The premix contained VA,1,900kU/kg; VD, 250kU/kg; VE, 3000 mg/kg; niacin, 4000 mg/kg; Cu, 1200 mg/kg; Fe, 525 mg/kg; Zn, 13,000 mg/kg; Mn, 5500 mg/kg; I, 170 mg/kg; Co, 50 mg/kg; Se, 27 mg/kg

^3^ The calculated nutritional composition values

### Sample collection

Cows were milked at 05:00, 13:00, and 21:00 h, and milk yield was recorded daily. Milk samples were collected to measure the somatic cell count (SCC) (MilkoScan FT1, FOSS, Denmark) once a week. Ruminal fluid samples were collected via the ruminal fistula for 3 consecutive days during the 18th week, at 2-h intervals starting at 04:00 h (after the morning feeding) for 12 h. The collected ruminal fluid samples were filtered through 2 layers of gauze. Rumen fluid samples were centrifuged at 10,000 × g for 45 min, and then the supernatant was aspirated and passaged through a disposable 0.22-μm filter. The filtrate was collected in a sterile, depyrogenated glass tube (previously heated at 250 °C for 2 h) and heated at 100 °C for 30 min. Samples were cooled at room temperature for 15 min and stored at—20 °C for subsequent LPS measurements. Blood samples were collected at 4 h after feeding on the sampling day of the 18th week. The samples were obtained via the lacteal artery and vein with 5-mL vacuum tubes containing sodium heparin. Samples were kept on ice until transported to the laboratory. Plasma was isolated from blood samples by centrifugation at 1469× *g* at 4 °C for 15 min and then stored at −20 °C for LPS, enzyme, and cytokine analyses. Mammary gland tissue samples were obtained by biopsy on the sampling day from the same quarter of the mammary gland [19]. Samples were rinsed with 0.9 % saline, fixed in 4 % (w/v) paraformaldehyde in PBS for immunohistochemistry, or snap-frozen in liquid nitrogen and then stored at −70 °C for RNA and protein extraction. Incisions were sutured, and antibiotics were administered intramuscularly to avoid infection.

### Ruminal pH, ruminal and plasma LPS

The ruminal pH values were measured with a pH metre immediately after collecting ruminal fluid. The concentration of LPS in ruminal fluid (CE64406) and plasma (CE80545) samples was determined by a chromogenic endpoint assay (Chinese Horseshoe Crab Reagent Manufactory Co., Ltd., Xiamen, China) with a minimum detection limit of 0.05 EU/mL (ruminal liquid) or 0.01 EU/mL (plasma) according to the manufacturer’s instructions.

### Pro-inflammatory enzyme activity and cytokine concentrations

The analyses of the myeloperoxidase (MPO) and β-N-acetyl glucosaminidase (NAG) concentrations were performed using commercial kits (Jiancheng Bioengineering Institute of Nanjing, Nanjing, China) that used an enzymatic colorimetric detection method read by a microplate reader (Epoch, BioTek, USA) [29]. Plasma IL-1β, IL-6, and TNF-α concentrations were determined using commercial radioimmunoassay kits (Beijing North Institute of Biological Technology, Beijing, China) and a gamma radioimmunoassay counter (SN-6105, Hesuo Rihuan Photoelectric Instrument Co., Ltd., Shanghai, China). All of the procedures were performed according to the manufacturer’s instructions.

### Reverse transcription and quantitative PCR

Total RNA was extracted from 100 mg of mammary gland tissue samples using RNAiso Plusreagent (TaKaRa Co., Otsu, Japan) according to the manufacturer’s protocol. The quantity and quality of the total RNA were assessed by a spectrophotometer (Eppendorf Biotechnology, Hamburg, Germany). The first-strand cDNA was synthesized using 250 ng of the total RNA template and a PrimeScript RT Master Mix Perfect Real Time Kit (TaKaRa Co., Otsu, Japan). The primers for the target genes were designed based on known cattle sequences using Primer Premier Software 5.0 (Premier Biosoft International, USA);these primers are shown in Table 2. The qPCR was performed on an ABI 7300 system (Applied Biosystems, Foster City, CA, USA) using a SYBR Premix Ex TaqKit (TaKaRa Co., Otsu, Japan) according to the manufacturer’s instructions. The 2^-ΔΔCt^ method was used to analyse the qPCR data, and glyceraldehyde-3-phosphate dehydrogenase (GAPDH) was used as an internal reference.

**Table 2:** The primer sequences of target and internal reference genes used in qPCR

Gene	Forward primer	Reverse primer	GenBank accession	PCR products
LAP	AGGCTCCATCACCTGCTCCTT	CCTGCAGCATTTTACTTGGGCT	AY911374.1	183 bp
BNBD5	CTGGATCACCTGCTCCTCGT	GGTGCCAATCTGTCTCATGTTG	NM_001130761.1	150 bp
IL-1β	GGCCAAAGTCCCTGACCTCT	CTGCCACCATCACCACATTC	NM_174093.1	167 bp
IL-6	GGAGGAAAAGGACGGATGCT	GGTCAGTGTTTGTGGCTGGA	NM_173923.2	227 bp
IL-8	CCTCTTGTTCAATATGACTTCCA	GGCCCACTCAATAACTCTC	NM_173925.2	170 bp
TNF-α	CTTCTGCCTGCTGCACTTCG	GAGTTGATGTCGGCTACAACG	AF_348421.1	156 bp
NF-kB	ATACGTCGGCCGTGTCTAT	GGAACTGTGATCCGTGTAG	NM_001045868.1	129 bp
GAPDH	GGGTCATCATCTCTGCACCT	GGTCATAAGTCCCTCCACGA	XM_001252479	177 bp

### Immunohistochemical analysis of LAP

Mammary gland tissue samples for immunohistochemical analysis were fixed in 4 % (w/v) paraformaldehyde in PBS, dehydrated and processed for paraffin sectioning (4 μm thick). Oven-dried sections were deparaffinised, followed by antigen retrieval by microwaving in EDTA buffer, and treated with 3 % H_2_O_2_ for 10 min to block the endogenous peroxidase. The sections were washed in PBS after each step. Then, the air-dried sections were blocked within 5 % BSA for 20 min followed by incubation with rabbit anti-LAP antibody (kindly provided by Isobe N [17], 2 mg/mL) in PBS with 0.5 % bovine serum albumin (BSA) overnight at 4 °C. After the sections were washed in PBS, they were incubated with HRP-conjugated secondary antibody using a REAL EnVision +/HRP Rabbit/Mouse Kit (Dako, Denmark) for 50 min at 4 °C, followed by washing in PBS. The products were visualized by incubating the sections with a diaminobenzidine (DAB) reaction mixture. The sections were then counterstained with haematoxylin, dehydrated and covered. Images were recorded by Nikon H550Smicroscope and NIS Elements F 3.0 software.

### Western blot analysis

Total protein from 100 mg of mammary gland tissue was extracted using RIPA lysis buffer (Beyotime, Shanghai, China), and the concentration was determined using a bicinchoninic acid protein assay (BCA) kit (Pierce, Rockford, IL, USA). The proteins were fractionatedby sodium dodecyl sulphate-polyacrylamide gel electrophoresis (SDS-PAGE) and then transferred onto nitrocellulose membranes (Bio Trace, Pall Co., USA). After the membranes were blocked with 5 % BSA at room temperature for 2 h, they were incubated with anti-LAP (1 μg/mL), anti-phospho-NF-kB p65 (Ser536) (Beyotime,Shanghai, China, 1:1000), or anti-phospho-IkB-α (Ser36/Ser32) (Signalway Antibody, LLC., Maryland, USA, 1:1000) at 4 °C overnight. Then, the membranes were incubated with horseradish peroxidase (HRP)-conjugated secondary antibody (E030130-01, Earth Ox, CA, 1:10000) at room temperature for 1 h. Antibodies directed against β-tubulin (Santa Cruz Biotechnology, Inc., 1:200) and GAPDH (Beyotime, Shanghai, China, 1:500) were used as internal controls for normalization. The membrane was washed, and the target proteins were visualized by enhanced chemiluminescence (ECL) using a BeyoECL Plus Kit (Beyotime, Shanghai, China). ECL signals were recorded by an imaging system (Fujifilm, Japan) and analysed with Quantity One software (Bio-Rad, USA). The membranes containing phospho-NF-kB p65 and phospho-IkBα were stripped by stripping buffer (Boster Biotechnology, Inc., Wuhan, China) according to the manufacturer’s instructions and incubated with antibodies directed against NF-kB p65 and IkB-α (Beyotime,Shanghai, China, 1:500), respectively. Then, the steps were followed as described above.

### Statistical analysis

The data of LPS concentrations of the rumen and lacteal artery and vein, as well as the concentrations of pro-inflammatory enzymes and cytokines in the lacteal vein plasma, were analysed using a MIXED model with repeated measures using SPSS Statistics (IBM, Inc., New York, USA). The data of the rumen pH values, the mRNA and protein expressions were analysed using the paired *t*-test. The correlations between the LAP mRNA expression and the protein expression were analysed using Pearson’s model. The data are expressed as the mean ± SEM. Differences were considered statistically significant if *p* < 0.05 or highly significant if *p* < 0.01, and a tendency was considered if 0.05 < *p* < 0.10.

## Results

### Ruminal pH and LPS content

The mean pH value of ruminal fluid in the HCD group was lower than that in the LCD group (*p* < 0.05) and was lower than 5.6 for more than 3 h per day (Table 3).

**Table 3:** Ruminal pH and LPS content in rumen samples and in plasma samples from the lacteal artery and vein

Item	Treatment		SEM	*p* value
	LCD	HCD		
Mean pH value	6.02	5.90	0.03	0.03
Time < pH 5.6, h/d	1.65	3.72	0.50	0.01
LPS in rumen, kEU/mL	47.17	79.04	7.94	<0.01
LPS in plasma of lacteal artery, EU/mL	0.47	0.86	0.06	<0.01
LPS in plasma of lacteal vein, EU/mL	0.12	0.27	0.03	<0.01

The LPS concentration in rumen samples was higher in the HCD group than that in the LCD group at 4 h after feeding (*p* < 0.01). The plasma LPS concentrations in the lacteal artery and vein increased in the HCD group compared with that in the LCD group (*p* < 0.01 for both, Table 3).

### Evaluation of MPO activity, NAG activity and pro-inflammatory cytokine concentrations

**Fig. 1:**
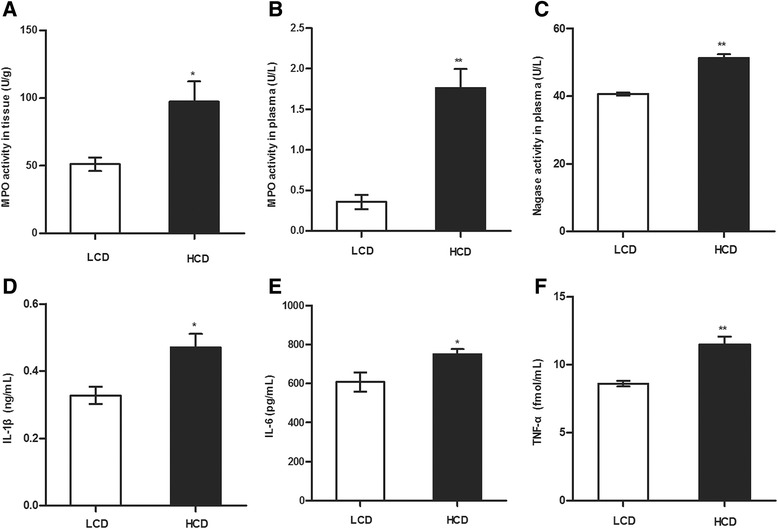
Effect of a HCD on MPO activity, NAG activity, and pro-inflammatory cytokine concentrations. **a** MPO activity in the mammary gland tissue. **b** MPO activity in the lacteal vein plasma. **c** NAG activity in the lacteal vein plasma. **d** IL-1β concentration in the lacteal vein plasma. **e** IL-6 concentration in the lacteal vein plasma. **f** TNF-α concentration in the lacteal vein plasma. Significant difference is indicated (*p < 0.05, **p < 0.01)

The MPO activity in the mammary tissue and the lacteal vein plasma in the HCD group was up-regulated compared with the LCD group as shown in Fig. 1a and b (*p* < 0.05 and *p* < 0.01, respectively).

The NAG activity in the lacteal vein plasma in the HCD group was higher than that in the LCD group (Fig. 1c, *p* < 0.01).

The concentrations of pro-inflammatory cytokines (IL-1β, IL-6, and TNF-α) in plasma from the lacteal vein increased in the HCD group compared with the LCD group (Fig. 1d, e, f, p < 0.05 for IL-1β and IL-6, *p* < 0.01 for TNF-α).

### Expression of immune-related genes in the mammary gland

**Fig. 2:**
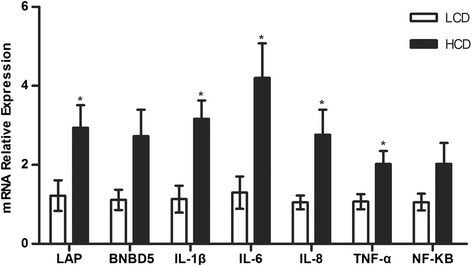
Quantitative real-time PCR. Quantification of the mRNA expression involved in immune and inflammatory responses in mammary glands of dairy cows fed a LCD or HCD. Single asterisk (*) indicates *p* < 0.05

The mRNA analysis showed that the expression of LAP, IL-1β, IL-6, IL-8, and TNF-α increased in the HCD group compared with the LCD group (*p* < 0.05, Fig. 2). However, no significant difference but a up-regulated tendency for BNBD5 expression was observed (Fig. 2). The expression of the transcription factor NF-kB, which is implicated in regulating inflammatory and immune responses, did not differ between the HCD group and the LCD group (Fig. 2).

### Immunohistochemical analysis of LAP

**Fig. 3:**
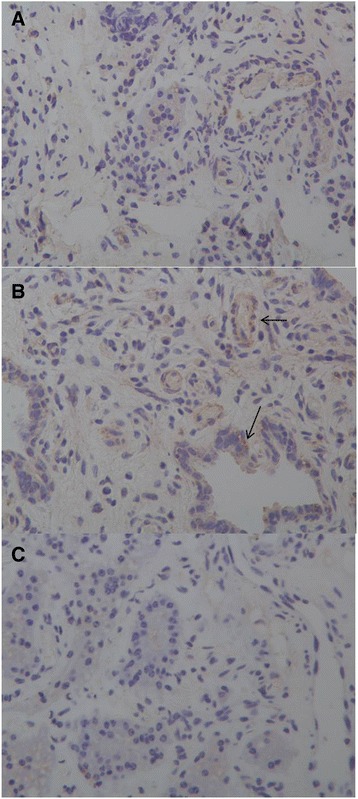
Sections of mammary gland immunostained for LAP (×400). **a** Alveolar epithelial cells from the LCD group that are immunonegative for LAP. **b** Alveolar epithelial cells from the HCD group that are immunopositive for LAP. Arrows show positive immunoreactions for LAP. **c** Alveoli cultured with normal rabbit IgG as controls for LAP

Positive immunoreactions of LAP in the HCD group were observed in the cytoplasm of mammary gland alveolar epithelium cells (Fig. 3b). Sections from the LCD group and the control sections incubated with normal rabbit IgG, which was substituted for LAP antibody, did not show positive reactions (Fig. 3a and c).

### LAP protein expression

**Fig. 4:**
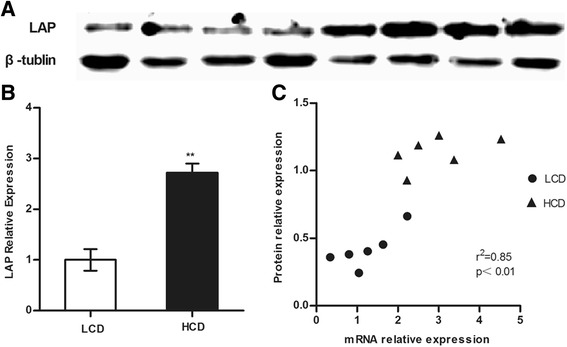
Effect of a HCD on LAP protein expression in mammary gland. **a** Western blot results of LAP and β- tubulin proteins (Bands 1-4 represent LCD group samples, while bands 5–8 represent HCD group samples). **b** The mean relative protein expression of LAP against the reference β-tubulin protein as quantified by band density. Significant difference is indicated (*p < 0.05). **c** Correlation between LAP mRNA and protein expression levels (*r*, coefficient of correlation (Pearson); *p*, significance of correlation)

LAP protein expression in mammary gland was up-regulated in the HCD group compared with that in the LCD group (Fig. 4a and b). In addition, individual relative LAP protein expression with its corresponding mRNA relative copy numbers showed a strong correlation (*r*^2^ = 0.85, *p* < 0.01, Fig. 4c).

### NF-kB signalling pathway analysis

**Fig. 5:**
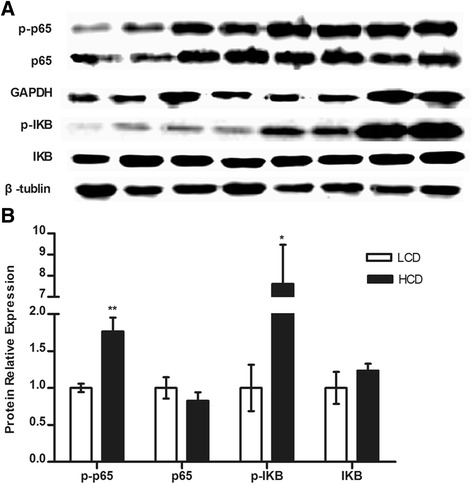
Effect of a HCD on the NF-kB signalling pathway in mammary gland. **a** Western blot results of phospho-NF-kB p65, p65 (GAPDH as an internal control), phospho-IkB, and IkB (β- tubulin as an internal control) proteins. Bands 1–4 represent LCD group samples, while bands 5–8 represent HCD group samples. **b** The mean relative expression of the target protein against the reference protein as quantified by band density. Significant difference is indicated (*p < 0.05, **p < 0.01)

Although the expression of NF-kB and IkBα proteins did not differ between the HCD group and LCD group, the amounts of phosphorylated NF-kB and phosphorylated IkBα increased in the HCD group compared with that in the LCD group (*p* < 0.01, *p* < 0.05, respectively, Fig. 5).

## Discussion

The present study demonstrated that rumen-derived LPS caused enhanced inflammatory responses, immune responses and LAP synthesis in mammary glands of cows fed a HCD.

Ruminants are often fed a relatively high proportion of grains or easily fermentable carbohydrates to support high milk yields [30]. Although this type of diet can promote cost efficiency over the short term, it would do harm to ruminant health and welfare in the long term [1]. In our present study, two different diets (HCD and LCD) were designed and used based on practical experience. The high incidence of SARA, a common digestive disorder in many dairy herds, is associated with feeding ruminants a HCD [1, 3, 5, 30] and also the physical form of total mixed ratio (both forage and grain) [31, 32], because a HCD can cause a decrease in ruminal pH due to the accumulation of short-chain fatty acids including organic acids and volatile fatty acids (VFA) [1, 33]. As shown in Table 3, the mean pH value of ruminal fluid in the HCD group was lower than that in the LCD group (*p* < 0.05) and was lower than 5.6 for more than 3 h per day, which is consistent with previous studies and which indicated that SARA was successfully induced by the HCD [2]. Alterations in the ruminal environment appear to lead to Gram-negative bacteria lysis and subsequent release of large amounts of LPS into the ruminal fluid [3, 5]. In the present study, the concentrations of LPS in the ruminal fluid and in the plasma from the lacteal artery and the lacteal vein of the HCD group were significantly higher than were those in the LCD group (*p* < 0.01 for all three of them) as shown in Table 3. These results are consistent with previous reports [1, 3] and indicated that a higher level of LPS did translocate into the circulatory system across the epithelial barrier of the gastrointestinal tract in the HCD group.

LPS in the peripheral blood initiates inflammatory responses [3, 5] by activating some inflammation-related enzymes and by secreting several pro-inflammatory cytokines. MPO, which is stored in azurophilic granules of neutrophils, is a neutrophil bactericidal protein. Its activity reflects the state of inflammation and oxidative stress [34]. NAG, one of the enzymes responsible for the metabolism of proteoglycans, is a good marker for estimating the degree of infection in bovine mammary glands [35]. Pro-inflammatory cytokines (IL-1β, IL-6, and TNF-α) play crucial role in inflammation [36]. In the current study, MPO activity, NAG activity, and pro-inflammatory cytokine (IL-1β, IL-6, and TNF-α) concentration in plasma from the lacteal vein in the HCD group increased compared with those in the LCD group, which provided evidence for activation of systemic inflammatory responses.

A high concentration of LPS enhanced the expression of various cytokines and β-defensins in the MECs of dairy cows [37]. MECs are located at the interface between the body and the environment. These cells not only function as a physical barrier for pathogens but also participate in the innate immune defence via producing inflammatory mediators such as cytokines, chemokines, and host defence peptides [38]. In the present study, the mRNA analysis of genes expressed in the mammary gland that are associated with inflammatory responses and immune responses, including LAP, IL-1β, IL-6, IL-8, and TNF-α, were all enhanced in the HCD group compared with that in the LCD group. However, BNBD5 mRNA expression was not significantly different but had an increasing tendency probably owing to the high variability between individual values. The LPS concentration in the lacteal vein in the HCD group might explain why the inflammatory and subsequent immune responses were activated in the mammary gland.

Not only the mRNA analysis but also the protein analysis of LAP showed a difference between the HCD group and the LCD group. LAP secretion in the cytoplasm of the MECs was demonstrated previously by immunohistochemistry [17]. In the present study, positive immunoreactions of LAP were observed in the cytoplasm of MECs in sections obtained from the HCD group, while the sections from the LCD group and the control sections did not show positive reactions. Furthermore, the western blot results confirmed that the LAP protein concentration in the HCD group was higher than that in the LCD group and had a strong correlation with its corresponding relative copy numbers of mRNA. Additionally, the finding that the SCC and the LAP protein concentration both increased in the HCD group compared with those in the LCD group is consistent with Kawai et al [39] and Isobe et al [40], who found that the SCC is associated with the LAP protein concentration in the milk. Recently, Kawai et al [41] also found that LF concentration decreased with decrease in SCC after antibiotics treatment, but not LAP concentration and LPO activity, which differed depending on the severity of mastitis.

The p65 subunit of NF-kB is known to play a stimulatory role in LAP expression in MECs. NF-kB can be activated by LPS and is implicated in regulating immune responses [42]. Mature NF-kB, which is sequestered in the cytoplasm in a quiescent state by interaction with IkBα, is a heterodimer of two subunits, p50 and p65, and the major pathway leading to their activation is based on IkBα phosphorylation and degradation [43]. The p50/p65 heterodimer translocates into the nucleus and binds to the kB sites on the promoter region of genes when the nuclear localization signal is exposed [44]. As shown previously, although NF-kB mRNA expression and NF-kB and IkBα protein expression did not differ between the HCD group and LCD group, phosphorylated NF-kB and phosphorylated IkBα protein expression significantly increased in the HCD group compared with that in the LCD group, which indicated that the NF-kB signalling pathway was activated after HCD administration. This result was consistent with previously reported results [24], thus we supposed that activation of the NF-kB signalling pathway is one of the mechanism that enhances LAP synthesis and immune responses.

## Conclusions

In summary, our study clearly documented that after long-term HCD feeding, rumen-derived LPS translocated to the blood stream, triggers inflammatory and immune responses and enhances LAP synthesis via the NF-kB signalling pathway in mammary glands of lactating cows. Our findings may shed light on the mechanism involved in rumen-derived LPS on LAP synthesis and immune responses in mammary glands of dairy cows fed a HCD.

## Abbreviations

BCA, bicinchoninic acid protein assay; BNBD, bovine neutrophil β-defensin; BSA, bovine serum albumin; DAB, diaminobenzidine; ECL, chemiluminescence; GAPDH, glyceraldehyde-3-phosphate dehydrogenase; HCD, high-concentrate diet; HRP, horseradish peroxidase; IkB, inhibitory kappa B α; IL, interleukin; LAP, lingual antimicrobial peptide; LCD, low-concentrate diet; LPS, lipopolysaccharide; MECs, mammary epithelial cells; MPO, myeloperoxidase; NAG, β-N-acetyl glucosaminidase; NF-kB, nuclear factor-kappaB; SARA, subacute ruminal acidosis; SCC, somatic cell count; TNF-α, tumour necrosis factor-α
